# Needs Assessment Towards Development of an Integrated Diabetes-Oral Health Nutrition Education Module: A Qualitative Study

**DOI:** 10.5334/ijic.5952

**Published:** 2021-09-15

**Authors:** Nor Aini Jamil, Shahida Mohd-Said, Chau Shin Hwa, Mohd Jamil Sameeha, Estie Kruger

**Affiliations:** 1 Centre for Community Health Studies (ReaCH), Faculty of Health Sciences, Universiti Kebangsaan Malaysia, 50300 Kuala Lumpur, Malaysia; 2 Department of Restorative Dentistry, Faculty of Dentistry, Universiti Kebangsaan Malaysia, 50300 Kuala Lumpur, Malaysia; 3 School of Human Science, Faculty of Science, The University of Western Australia, 6009 Perth, Australia

**Keywords:** hyperglycaemia, periodontitis, health literacy, in-depth interview, healthcare professional

## Abstract

**Introduction:**

The diabetes mellitus-periodontitis relationship has advocated several strategic management approaches on effective glycaemic control and oral health improvement for diabetic patients and periodontitis patients with diabetes. We aimed to identify the challenges of providing health education to patients with diabetes and/or periodontitis among healthcare professionals and needs for an integrated nutrition-oral health education module.

**Methods:**

This study involved semi-structure in-depth interview with fifteen healthcare professionals from a training hospital focused on: (i) the existing issues and challenges encountered while managing patients for their nutrition and care and (ii) issues related to the current practice among healthcare professionals. Details pertaining to the participants’ verbal and non-verbal responses were recorded, transcribed ad verbatim and analysed using themes codes.

**Results:**

Patients’ attitude and behaviour, language barriers and prioritising time were found as the common problems with patients, while limited knowledge on the relationship between diabetes-periodontitis, limited availability of appropriate and cultural-based health educational tools, lack of inter-professional multidisciplinary collaboration in managing patients, and constrains in time as well as costly therapy were common issues in the current practice.

**Conclusions:**

Cost-effective efforts must be focused on overcoming these issues besides emphasizing the needs on developing an integrated module to achieve better management outcomes.

## Introduction

The bidirectional relationship between type II diabetes mellitus (T2DM) and periodontitis has established that not only is periodontitis is a complication of diabetes mellitus [1] and periodontitis severity is worsened by diabetes [2], improvement of glycaemic status and control of periodontitis progression via dental treatment and oral health improvement also provide beneficial clinical outcomes of both diseases [3,4,5]. Undoubtedly, the complications of these chronic diseases have significant impacts on the wellness and quality of life of the patients [6,7,8], the healthcare services and also becomes an economic burden to a nation [9,10,11,12]. However, studies have shown that the risk of diabetes complications can be minimized if glycaemic control is maintained within the normal range (3.9–7.2 mmol/l) [13] by disseminating knowledge related to self-care to patients, as well as professional intervention via periodic supportive treatment [4,12,14,15,16]. This fundamental breakthrough hence has advocated several recommendations by both endocrinologists and periodontists for concurrent glycaemic control and oral health education with professional dental care as the combined strategic approach for managing the disease [4,12,17].

It is acknowledged that awareness of periodontitis and interactions between diabetes and periodontitis is relatively low among diabetic patients [18,19], while many still perceive that dental check-ups are not important [20,21,22]. More importantly, healthcare professionals with non-dental training are equally unaware of the link between both diseases, hence do not see the importance of referring patients for dental check-ups [20,21,22]. Although the existing medical and health education show limited input on oral health education through the training of future healthcare professionals [23,24], efforts have been made to incorporate oral health education into the allied health sciences curriculum and some multidisciplinary collaborative teams [25,26,27].

Notable improvements of glycaemic control and quality of life have also been evident following health and nutrition education programmes provided to patients with diabetes [28,29,30,31,32,33,34,35]. In oral health management, particularly for treatment of periodontitis, there has been discussion on the possible association between the role of nutrition and the disease severity [12,36,37,38]. However, limited studies are currently available regarding the effectiveness of nutrition education programs on periodontitis therapy, while available modules only have specific information on individual diseases and education tips focused on either diabetes or oral health care alone [39,40]. The inclusion of nutrition and oral health education aimed at patients with T2DM and in glycaemic control of periodontitis patients with diabetes is crucial to ensure a comprehensive understanding of the relationship between both diseases, as well as an emphasis on their treatment benefits in the control of disease progression. Nevertheless, understanding of the existing issues and challenges faced by healthcare professionals when providing the health education module is firstly required and the design and content of the module has to be proposed accordingly. Hence, this study aimed to identify the challenges of providing health education to patients with diabetes and/or periodontitis among healthcare professionals and needs for an integrated nutrition-oral health education module.

## Materials and methods

The participants of this study were recruited among healthcare professionals from a public university hospital in Kuala Lumpur, with a minimum of 3-years’ experience in diabetes or periodontitis care. A purposive sampling technique [41] was employed to include healthcare professionals who are currently serving the primary health clinic, and/or dealing directly with diabetes and periodontitis patients in their respective clinics and deemed suitable for the task recommended by their respective Heads of Departments. All participants were provided with the study information and gave their written informed consent to take part. Ethics approval was obtained from the Research Ethics Committee (reference: PPI/111/8/JEP-2019–115) prior to the study commencement.

Data collection was carried out through semi-structured in-depth interviews with each participant in both English and Malay languages by a trained interviewer (C.S.H.), either in private office rooms, diet counselling clinics, or dental clinics at the convenience of the participants. The interview questions focused on: (i) the existing issues and challenges encountered while managing diabetes and/or periodontitis patients in relation to their nutrition and care and (ii) issues related to the current practice among healthcare professionals (***Table 1***). In the process, participants were probed to further elaborate on their answers until all data reached saturation i.e., no new information was obtained. The average duration of each interview session amounted to 45 minutes.

**Table 1:** Interview questions for needs assessment of a new integrated nutrition module.


**I. Based on your experience in management glycaemic control/periodontitis *(choose one):*** what do your patients usually complain about? how do you handle these complaints? what is your biggest concern in the management of glycaemic control/periodontitis (choose one)? what are your management strategies to improve patients’ glycaemic control/oral health (choose one)? what are the common problems or challenges you encounter when educating/counselling patients with T2DM/periodontitis (choose one)? what are the misconceptions regarding T2DM/oral health (choose one) that you usually hear from patients? how do you explain or clarify those misconceptions to your patients?
**II. In your routine management of patients, do you:** educate the patients about the risk of having periodontitis/uncontrolled diabetes (choose one)? get referral from a health professional/dentist(choose one) to manage their oral health/glycaemic condition (choose one)? use any educational tools during the education or counselling session? If NO, why not? If YES, What are the educational tools? Do you find the educational tools useful? Do you find them useful for patients to take home and use? Are there any limitations to the tools you are using?


A digital voice recorder (Sony ICD-PX470, Sony Corporation, Japan) and an Android Huawei mobile phone (Huawei Y9, Huawei Device Co., Ltd., China) were used to record all interviews, in addition to written field notes during the interview sessions which were later summarized. Details pertaining to the participants’ verbal and non-verbal responses such as body language, vocal tone, facial expressions, atmosphere, and any special occasions that emerged during the interview sessions were also recorded as they might have influenced the validity of the findings.

Next, the interview data were transcribed ad verbatim while transcripts generated were checked in comparison with the original audio files to identify any missing information and followed by thematic analysis [42]. Two other researchers, as experts in Nutrition Education (N.A.J. and M.J.S.), were tasked to check the contents and initial codes based on the interview objectives, whereas the subsequent coding process was completed using NVivo 12 Plus software programme. Here, different codes were sorted into potential categories and themes, accordingly, using the techniques recommended by Braun, Clarke, & Terry [42]. The themes obtained were reviewed by reading through all of the collated extracts for each theme to ensure that the data within them showed a coherent pattern. Finally, the themes were defined and refined, while some of the quotes required translation from Malay to English language to ensure the original meaning had been retained.

## Results

### Socio-demographic profiles

A total of 15 healthcare professionals (mean age 41.9 years, SD 8.0) consisted of dietitians, diabetic educators, dental professionals (dental specialist, dental officer, and dental nurse), an (endocrinologist), health psychologist, and pharmacist took part in the interviews. Majority of them were female (87%) with an average service duration of 15 years (SD 6.6) in the current health institution.

### Challenges in managing patients

Several challenges were reported in detail by the participating healthcare professionals when educating their patients, particularly patient’s attitude and behaviour towards prioritising their own health care (***Table 2***). These include patient’s refusal to make lifestyle changes, refusal to undergo advanced treatment, over-reliance on healthcare professionals, patient’s higher tendency to listen to their family members or peers, and preference to use alternative therapy instead of hospital-prescribed medication.

**Table 2:** Themes for challenges in managing patients with diabetes and/or periodontitis.


THEMES	SUBTHEMES	QUOTES

Patient’s attitude and behaviour towards own health care	Refusal to make lifestyle changes	*“Some of the patients came here because of request by the doctor. In reality, they do not want to change or accept making changes. They just don’t have that yet – the willingness to make changes. For me, that’s difficult. We can educate, we have (can provide) information and knowledge, but they just do not (want to) accept.” (HP 6)*

	Refusal to undergo advanced treatment	*“Another obstacle in (getting a) successful outcome, sometimes when the patients hear: “(dental) surgery? No need, doctor. The non-surgical procedure is sufficient, I don’t want to undergo surgery.” (HP 14)*

	Relying too much on healthcare professional	*“Some of the patients would just leave everything to the doctors and depend on whatever the doctor wants to do.” (HP 12)*

	Listening more toward family members and peers	*“The common problem is they listen more to their peers and family members, rather than the doctors.” (HP 12)*

	Preference for alternative medicine	*“There are a lot of challenges, especially their preference to alternative medicine such as supplements and traditional medicine. When they buy these kinds of things (i.e., medicine), there is a tendency wherein they will not use hospital medication. That is the biggest challenge.”* (*HP 13)*

Language barrier	Patients only speak their mother tongue	“*Usually (patients) some of them do not understand English, so they prefer (their own mother tongue) other language. It’s difficult. So, there’s a barrier.” (HP 2*) “*… especially among patients, we do not have that communication skill (i.e., to converse in their ethnic language). So, there’s a language barrier.” (HP 10*)

Prioritising time for counselling sessions	Patients in a hurry	*“Usually when the patients come to the hospital, they are in a rush. They don’t want to wait. When you talk to them, they try to push you to talk as fast as possible so that they can go back as early as possible. They don’t want to wait for long. That is the main barrier.” (HP 13)*


### Challenges and issues related to the current practice

Through the interviews, several common issues were voiced by the participants (***Table 3***) particularly regarding the health professionals’ knowledge and competencies including limited multi-language skills to communicate with the mixed ethnicity population, cost and time constraints, lack of integration in multidisciplinary care, limited available educational materials [43,44,45,46,47] for providing a cost-effective and continuous education module and limited cultural-based modules. Some participants highlighted the issue of common practices and consumption of local food related to certain culture and ethics. Hence emphasising the needs for providing health nutrition education in common local languages, in a simple way as possible to cater the difference in literacy level of readers within a diverse multi-ethnic population.

**Table 3:** Common issues related to management of diabetes and/or periodontitis.


THEMES	SUBTHEMES	QUOTES

Health professionals’ knowledge and competency	Limited number of health professionals with multi-language skills	“*We will refer to other pharmacists for different (non-Malay or English speaking) patients. … we have other diabetic educators also (who speak different languages)”* (*HP 4)*

	Low awareness of the relationship between diabetes and periodontitis	*“So far, people (i.e., healthcare professionals) just reinforce the microvascular complications such as the kidney, eyes and macrovascular complications like nerves and heart. Therefore, they don’t realize that there is another thing (i.e. oral health) that is also important. It is a small thing, but important.” (HP 3)*

		*“If you read everywhere, they talked about the macrovascular and microvascular complications. They don’t include this issue (i.e., periodontitis in their talks).” (HP 11)*

		*“Periodontitis… I’m not aware of it. Because periodontitis is not, I think at this moment, part of the assessment that the pharmacist needs to do.” (HP 13)*

		*“I think maybe dental students also should be taught to refer to the dietitian. Let say for example, the student noticed that the patient has some severe nutritional problems, maybe can be referred to the dietitian for counselling because the knowledge that the dental students or the dental professions have on nutrition is quite limited.” (HP 7)*

Lack integration in multi-disciplinary care	Conflicting messages in a multidisciplinary team	*“In terms of the multidisciplinary team, among the doctors, nurses, (and) dietitians, when they talk to the patient most of the time, they are not providing the same messages.” (HP 11)*

	Lack of referral and inter-professional collaboration	*“From my experience in handling patients, we don’t get any referral from the dentist. Also never get those with the periodontitis problems before. I can say that none of the patients will discuss that (i.e. periodontitis problems) and (how that can relate) to the diabetes management.” (HP 1)*

		*“We only have (referral) for the medical department. We don’t actually refer to the dietitian.” (HP 7)*

		*“Never. So far no (referrals from dental team). We only refer to other clinics. Dental has never referred to us (either).” (HP3)*

		*“When there’s a patient who complained to the doctor that he had a problem, only (then) we will make a referral to the dental department.” (HP 3)*

		*“I think maybe dental students should also be taught to refer to the dietitian. Let’s say, for example, the student notices that the patient has severe nutritional problems or maybe they can actually be referred to the dietitian for (dietary) counselling.” (HP 7)*

		*“And I also hope that medical practitioners, when conducting medical check-ups, will refer the patient for a dental check-up or treatment.” (HP 7)*

Limited available educational materials	Limited number and access to educational materials	*“Previously, I went to KKM (Ministry of Health) to collect (educational) materials. However, there is no more now. So, I just give advice verbally.”* (*HP 3*)

		*“I don’t have any specific module to give to the patients.”* (*HP 14*)

		*“We have pamphlets from … (industry). We give these for them (patients) to read.” (HP7)*

		*“I don’t have any specific module to give to patients.” (HP14)*

	Unattractive materials	*“So far, we only have black and white factsheets as coloured printing is costly*.” (*HP 10*)

		*“.. due to the cost, it’s better to be colourful… if in black and white, you cannot visualize (the diagrams or photos).”* (*HP 3*)

Limited cultural-based module	Diversity of culture and practices among the multi-ethic population	*“I think the component of culture, we need to put in something. …there are a lot of cultural things that need to be put in.” (HP13)*

		*“In Malaysia, we need to mix everything. But if you form (develop) a (new) module, you need to find the most common (eating) practice).” (HP11)*

		*“…must include the common local food.” (HP6)*

	Multi languages for multi-ethics	*“English and Malay could be better actually…” (HP3)*

		*“I think all these modules must be made with multiple languages. You must also consider other ethnic groups.” (HP14)*

		*“… the official language is Bahasa Malaysia (Malay language), so therefore, by right, everyone (is) supposed to understand it.” (HP1)*

Time constraint	Limited time for education and counselling session	“*Because our clinic has a lot of patients and we have limited time.” (HP 12*)

		“*I think that’s one of the reasons why sometimes the messages do not get across to the patients; because of the time factor; periodontists wouldn’t have much time to repeat these messages over and over again.” (HP 14)*

	Patients in a hurry to get home	*“Usually when the patients come to the hospital, they are in a rush. They don’t want to wait. When you talk to them, they try to push you to talk as fast as possible so that they can go home as early as possible. They don’t want to wait for long.” (HP 13)*

Costly therapy	Expensive treatment for complex and severe cases	*“Cost is again another barrier. To be able to get a good result in treatment, we need to up notch it to a more complex surgical phase. This may need more (incur a higher cost), which patients need to commit to in terms of financial and it is a lot. … don’t see that they should be committing (to) that amount of money just to retain one or two teeth.” (HP 14)*

	Cost for long term monitoring	*“it is not easy to tell patients to monitor their blood sugar level every day because of the cost involved.” (HP 12)*


## Discussion

Patient awareness, behaviour, attitude, and compliance to health advice are some of the utmost important aspects in ensuring success of health education modules for patients with diabetes as well as periodontitis. At the same time, it is fundamental to ensure that healthcare professionals are well-informed about the bidirectional relationship between T2DM and periodontitis and constantly updated with the current approaches of managing both diseases.

Compliance of patients in following nutrition and health advice as well as treatment regimens has been found to be one of the key factors for successful treatment in both T2DM and periodontitis management [22,48,49], and has been emphasized by most of the participating healthcare professionals in our study. Effective oral hygiene care, for instance, is especially important in periodontitis management and should be made part of the diabetic patient’s daily routine [50]. Moreover, extensive education regarding effective methods of plaque control and correcting any improper brushing techniques among diabetic patients are equally necessary due to their potential in improving one’s metabolic control [51,52]. From the perspective of diabetes management, patients should be empowered to capably monitor their blood sugar levels and be disciplined in committing to their lifestyle changes. Here, the consumption of nutrient-dense food such as fruits, vegetables, and whole grains products along with limiting high sugar content food items are beneficial in reducing the inflammation in periodontitis and improving one’s periodontal health [53]. Tips on eating out should also be included in the module to limit the consumption of energy-dense food when not eating at home, or eating home-prepared food, which are to be carried out together with regular physical activity and exercises to improve insulin resistance [54,55]. More importantly, the module should be developed in multi-languages in consideration of and to overcome language barriers among patients.

Among the most important issues identified in our study was the low awareness of the relationship between diabetes-periodontitis among healthcare professionals themselves. Other than the dental professionals, none of them had any dental education training and were unaware of the relationship between diabetes and periodontitis and reported to have never seen diabetic patients with periodontitis in their clinics. Meanwhile, dental professionals would only make referrals to the medical department for blood glucose management, and the health personnel only made referrals to the dental department, when diabetic patients complained of dental problems. This element of knowledge is utmost crucial to ensure that information on the relationship between diseases are being translated accurately and sufficiently to all patients with periodontitis and diabetes in the future nutritional and health education module, and that appropriate referrals are being made in timely manner.

The presence of conflicting messages within a multidisciplinary team and the lack of referral and inter-professional collaboration were also identified in our study. This could be one of the reasons leading to patient non-compliance with their disease management. To these, all the participants agreed to efforts in building a united collaborative approach among the multidisciplinary team in order to provide more comprehensive nutritional advice for patients with diabetes and periodontitis. They also supported their health and medical counterparts to refer patients for dental check-up and treatment for the prevention of oral diseases. In addition, the patients must be informed through consistent messages by all members of the healthcare professional team regarding the equally crucial role of disease management for both diseases [22,56] as they could affect one another as a result of the up-regulated inflammation from each disease.

The emergence of non-evidence-based remedies and uncertified products easily available in the market has posed a challenge for the delivery of conventional treatment prescribed by healthcare professionals [50,57]. Here, patients tend to believe in the former, thus potentially resulting in the manifestation of more complications due to the lack of their knowledge and awareness as opposed to obtaining information from authorized personnel or organization. Our study also identified the needs to highlight evidence-based treatment and the advice to seek help from authorized healthcare professionals. It is highly necessary for the development of a module geared toward filling such a gap in the current practice. Additionally, the myths and misconceptions commonly encountered about T2DM and periodontitis should also be emphasized to prevent any delays in receiving the proper treatment. This would further enhance patient’s adherence toward the appropriate treatment as planned by the healthcare professionals.

The strength of the present study is that this was the first in-depth, qualitative investigation that identified the challenges faced by healthcare professionals when providing nutrition and health education to patients with T2DM and periodontitis in Malaysia. This study also addresses the gap that exists in the management of T2DM and/or periodontitis and the needs to enhance awareness on the link between T2DM and periodontitis in patient care. Our study was based on fifteen healthcare professionals in a university hospital; hence the result of this study might not be generalisable to the entire healthcare professional workforce.

Overall, the participating healthcare professionals highly supported a more integrated, attractive, simple yet compact, and culture-appropriate nutrition-oral health education module to their patients with diabetes and periodontitis. Efforts must be focused on overcoming these issues besides emphasizing the needs on developing an integrated module to achieve better management outcomes.

## Conclusions

Patients’ attitude and behaviour, language barriers and prioritising time were found as the common problems with patients, while limited knowledge on the relationship between diabetes-periodontitis, limited availability of appropriate and cultural-based health educational tools, lack of inter-professional multidisciplinary collaboration in managing patients, and constrains in time as well as costly therapy were common issues in the current practice.
